# Prepatterning and patterning of the thalamus along embryonic development of *Xenopus laevis*

**DOI:** 10.3389/fnana.2015.00107

**Published:** 2015-08-10

**Authors:** Sandra Bandín, Ruth Morona, Agustín González

**Affiliations:** Faculty of Biology, Department of Cell Biology, University ComplutenseMadrid, Spain

**Keywords:** forebrain, diencephalon, zona limitans intrathalamica, Gbx2, Shh, Nkx2.2, evolution

## Abstract

Previous developmental studies of the thalamus (alar part of the diencephalic prosomere p2) have defined the molecular basis for the acquisition of the thalamic competence (preparttening), the subsequent formation of the secondary organizer in the zona limitans intrathalamica, and the early specification of two anteroposterior domains (rostral and caudal progenitor domains) in response to inducing activities and that are shared in birds and mammals. In the present study we have analyzed the embryonic development of the thalamus in the anuran *Xenopus laevis* to determine conserved or specific features in the amphibian diencephalon. From early embryonic stages to the beginning of the larval period, the expression patterns of 22 markers were analyzed by means of combined *In situ* hybridization (ISH) and immunohistochemical techniques. The early genoarchitecture observed in the diencephalon allowed us to discern the boundaries of the thalamus with the prethalamus, pretectum, and epithalamus. Common molecular features were observed in the thalamic prepatterning among vertebrates in which Wnt3a, Fez, Pax6 and Xiro1 expression were of particular importance in *Xenopus*. The formation of the zona limitans intrathalamica was observed, as in other vertebrates, by the progressive expression of Shh. The largely conserved expressions of Nkx2.2 in the rostral thalamic domain vs. Gbx2 and Ngn2 (among others) in the caudal domain strongly suggest the role of Shh as morphogen in the amphibian thalamus. All these data showed that the molecular characteristics observed during preparttening and patterning in the thalamus of the anuran *Xenopus* (anamniote) share many features with those described during thalamic development in amniotes (common patterns in tetrapods) but also with zebrafish, strengthening the idea of a basic organization of this diencephalic region across vertebrates.

## Introduction

The thalamus is a pivotal forebrain structure that serves as relay station for sensory information (except olfaction) to the overlaying cortex in mammals (Jones, 2007; Nieuwenhuys et al., 2007). Thus, it was described as the “gateway to consciousness” (Crick and Koch, 2003). Also in non-mammalian vertebrates, and particularly in anamniotes, the thalamus primarily relays sensory information to the telencephalon, although predominantly to subcortical (subpallial) regions (Marín et al., 1998; Laberge et al., 2008; Mueller, 2012).

Anatomically, the thalamus represents the largest part of the diencephalon. According to the current prosomeric model of forebrain development (Puelles and Rubenstein, 1993, 2003), once the brain regionalization starts the diencephalon soon subdivides into three segments or prosomeres that are defined by distinct gene expression patterns and morphological landmarks (Puelles and Rubenstein, 1993, 2003; Puelles, 1995, 2001; Puelles et al., 2004, 2012). The three segments are known as p1 (caudal, adjacent to the mesencephalon), p2 (intermediate) and p3 (rostral, adjacent to the secondary prosencephalon formed by the hypothalamus and telencephalon), each one containing roof, alar, basal and floor plate territories (Puelles and Martínez, 2013). The neuroepithelium in the alar diencephalon gives rise to the pretectum (in p1), the thalamus and epithalamus (in p2) and the prethalamus (in p3) (Puelles and Rubenstein, 2003).

Developmental studies have yielded important clues on how the different components of the mature thalamus are specifically determined by distinct combinations of gene expressions at particular times during development (Suzuki-Hirano et al., 2011). Thus, early in development a “prepatterning” phase was distinguished, in which the prospective thalamic territory in the diencephalon acquires its competences (Puelles and Martínez, 2013). Dorsoventral prepatterning is guided mainly by gradients of dorsally secreted morphogens like members of the Wnt and Bmp families, whereas Sonic hedgehog (Shh) is the leader morphogen secreted ventrally (Briscoe, 2009). In this period, it was demonstrated in different species that antero-posterior interactions of genes expressed rostral to the p3/p2 boundary (Six3, Fezf) and those genes expressed caudal to it (Irx, Otx) seem to demarcate a particular region where a most essential structure for thalamic development is formed, the *zona limitans intrathalamica* (Zli), also known as mid-diencephalic organizer (Kobayashi et al., 2002; Hirata et al., 2006; Scholpp et al., 2007; Scholpp and Lumsden, 2010). This represents a secondary organizer between the thalamus and prethalamus that releases several secreted signaling factors, including Shh, and members of the Wnt and fibroblast growth factor (Fgf) family (Bulfone et al., 1993; Echevarría et al., 2003; Kiecker and Lumsden, 2004; Vieira et al., 2005; Zeltser, 2005; Hagemann and Scholpp, 2012). Numerous previous works have shown that Shh is the main secreted molecule of the Zli that influences patterning of the thalamus and prethalamus in all vertebrates studied so far (Hashimoto-Torii et al., 2003; Kiecker and Lumsden, 2004; Vieira et al., 2005; Hirata et al., 2006; Scholpp et al., 2006; Guinazu et al., 2007; Szabó et al., 2009; Epstein, 2012).

During the subsequent “patterning” phase of the thalamus, two distinct progenitor domains are formed, primarily in response to Shh secreted from the Zli (and basal plate), that are distinguishable by molecular markers (Jeong et al., 2011; Suzuki-Hirano et al., 2011). A small rostral region occupies the rostroventral part of the thalamus (rostral thalamus, r-Th) and seems to be formed under the combined influence of high levels of Shh secreted from the Zli and the basal plate. Therefore, both anteroposterior and ventrodorsal signaling specified this region (also named anterobasal domain; Puelles and Martínez, 2013). The caudodorsal part of thalamus (caudal thalamus, c-Th) is a much larger region and is gradually exposed to lower amounts of Shh. The high concentration of Shh that reaches the r-Th makes the progenitor cells in this region to express Nkx2.2, Ascl1 (Mash1) that finally leads to the GABA phenotype of thalamic neurons (Vue et al., 2007; Chatterjee and Li, 2012; Robertshaw et al., 2013). In turn, progressively less Shh in the c-Th induces expression of different genes such as Gli1/2, Ngn1/2, Lhx9, Dbx1, Gbx2, and finally leads to the differentiation of the glutamatergic thalamic neurons (Hashimoto-Torii et al., 2003; Kiecker and Lumsden, 2004; Vue et al., 2007, 2009; Kataoka and Shimogori, 2008; Chatterjee and Li, 2012).

Comparative studies on the gene expression patterns along thalamic development during prepatterning and patterning have demonstrated a basically identical sequence in chicken and mouse (Scholpp and Lumsden, 2010; Martinez-Ferre and Martinez, 2012; Puelles and Martínez, 2013; Robertshaw et al., 2013). Moreover, recent studies in zebrafish have shown readily comparable gene expression patterns during early thalamic development (Scholpp and Lumsden, 2010; Hagemann and Scholpp, 2012).

Neuroanatomical and developmental studies of amphibians are interesting because they are the only group of tetrapods that are anamniotes, and constitute a key model for the understanding of the anamnio-amniote transition, since they share characteristics with amniotes (reptiles, birds, and mammals) and other anamniotes. Interestingly, the analysis of the genoarchitecture has been revealed as a powerful tool in the identification the regions in the amphibian brain that are homologous to those with similar genetic features in other vertebrate groups (Bachy et al., 2001, 2002; Brox et al., 2003, 2004; Moreno et al., 2004, 2008a,b, 2012b; Domínguez et al., 2010, 2013, 2014; Morona et al., 2011; Bandín et al., 2013, 2014; Joven et al., 2013a,b). In amphibians, the thalamus was classically considered to be part of the *pars dorsalis thalami* that often contained most pretectal structures (Herrick, 1933), and this interpretation was largely maintained in subsequent studies (Neary and Northcutt, 1983). More recently, Puelles et al. (1996) applied the prosomeric model to the interpretation of the anuran diencephalic cell groups, and described the thalamus (formerly the dorsal thalamus) as a distinct neuromeric alar region in p2. In the present study, we aim to analyze the organization of the thalamus along the embryonic development in the anuran amphibian *Xenopus laevis*, by studying the sequential expression patterns of a number of genoarchitectonic markers correlative with those that are significant in the formation of the thalamus of other vertebrates, primarily mammals. This set of markers included genes specifically involved in thalamic development, such as Shh, Wnt3a, Fez, Pax6, Xiro1, Nkx2.2, Dbx1, Gbx2, Gli1, Gli3, Gli4, Lhx2/9, Ngn2, Tcf4, and Vglut2. In addition, we studied the distribution of other markers for regions adjacent to the thalamus and that help in the localization of thalamic boundaries in *Xenopus*, such as Dll4, Emx1, Lhx1, GABA, Islet 1(Isl1), and Pax7 (Moreno et al., 2004, 2008a,b; Domínguez et al., 2010, 2011; Morona et al., 2011; Bandín et al., 2013, 2014). The results have been analyzed in relation to the current prosomeric model (Puelles and Rubenstein, 2003), which provides an important framework for establishing evolutionary conserved maps with corresponding gene expression relationships in the vertebrate neural tube. The detailed molecular characterization observed in early development of *Xenopus* embryos strongly indicates molecular conservation during thalamic development in vertebrates.

## Materials and Methods

### Animals and Tissue Processing

The original research reported herein was performed according to the regulations and laws established by European Union (2010/63/EU) and Spain (Royal Decree 53/2013), after approval from the Complutense University to conduct the experiments described. For the present study, a total of 371 embryonic *Xenopus laevis* specimens were used (Table 1).

**Table 1:** **Number of animals used in each experimental approach**.

	ISH		ISH + IHC		IHC		Total
	ISH simple	ISH double	ISH + IHC	FISH + IHF	IHC simple	IHF double
**Embryos**	20	45	210	60	24	12	**371**

*Xenopus laevis* embryos were obtained by human chorionic gonadotropin (HCG)-induced egg-laying followed by *in vitro* fertilization. Adult frogs were purchased from the CNRS colony (Montpellier or Rennes, France). Embryos were selected and staged according to Nieuwkoop and Faber (1967) into embryonic (33–45) stages. At appropriate times, they were deeply anesthetized by immersion in a 0.4mg/ml solution of tricaine methanesulfonate (MS222, Sigma, St. Louis, MO, USA), pH 7.4. The embryos were fixed by immersion in 4% paraformaldehyde in 0.1 M phosphate buffer (PB, pH 7.4) or in MEMFA (1 M 3-([*N*-morpholino]) propanesulfonic acid [MOPS](Sigma-Aldrich), 20 mM EGTA (Sigma-Aldrich), and 10 mM magnesium sulfate in 4% formaldehyde, adjusted to pH 7.4.) fixative solution overnight at 4°C. They were then processed *in toto* for *In situ* hybridization (ISH) or Immunohistochemistry (IHC) and finally sectioned on a freezing microtome.

### *In Situ* Hybridization (ISH)

The embryos were processed for ISH with several digoxigenin-UTP-labeled probes and with fluorescein-UTP-labeled (Shh) antisense riboprobes (Table 2). The plasmids were linearized with the appropriate restriction enzymes (Promega, Madison, WI, USA) and used as templates for RNA synthesis with T3, T7 or SP6 polymerase (Promega; Table 2).

**Table 2:** **List of gene markers used, sequence of the gene in the construct, origin of the plasmid, and enzymes employed to synthesize the probe**.

Gene	Genebank Acc. No.	Origin	Linerization restriction/polymerase
**Gbx2**	L47990	Dr. D. Kimelman, Dept. Biochemistry, Univ. Washington, Seattle, WA 98195-7350	HindIII/T3
**Xiro1**	AJ001834	Dr. Jose Luis Gómcz-SkarmeLa, Centro Andaluz de Biologia del Desarrollo CSIC/Universidad Pablo de Olavide, Spain	EcoRI/T7
**Tcf4 (Tcf7L2)**	HQ121418	Morona et al. (2011)	Pst1/T7
**Shh**	NM001088313	Dr. Randal Moon, University of Washington, Seattle	BamHI/T3
**Lhx1**	NM001090659	Dr. Sylvie Rétaux, CNRS, Paris, France	Ncol/SP6
**Lhx9**	AJ311711	Dr. Sylvie Rétaux, CNRS, Paris, France	Ncol/SP6
**Lhx2**	AJ311713	Dr. Sylvie Rétaux, CNRS, Paris, France	Ncol/SP6
**Ngn2**	U67779	Bellefroid, Institute of Biochemistry and Molecular Cell Biology, Göttingen, Germany	BamH1 /T3
**Dbx1**	AF253504	Dr. Sylvie Rétaux, CNRS, Paris, France	SaII/T7
**Dll4**	NM001090563	Nancy Papalopulu, Manchester University, Manchester	NotI/T3
**Gli1**	U57454	Takabatake T, Furo-cho, Chikusa-ku, Nagoya, Japan	BamHI/T7
**Gli3**	U42461	Takabatake T, Furo-cho, Chikusa-ku, Nagoya, Japan	BamHI/SP6
**Gli4**	U42462 (clone described as Gli2: AF109923)	Takabatake T, Furo-cho, Chikusa-ku, Nagoya, Japan. Ruiz i Altaba. NYU Medical Centre, NY, USA	NotI/T7
**Vglut2**	NM_00109161.1	J. L. Ferrán /R. Morona, University of Murcia, Murcia, Spain	NcoI/SP6
**Wnt3a**	M55054	Dr. Randal Moon, University of Washington, Seattle	ClaI/T7
**Emx1**	NM_001093430.1	Dr. Sylvie Rétaux, CNRS, Paris, France	HindIII/T7
**Fez**	AF195021	Sargent TD, Department of Health and Human Services, Bethesda, Maryland	Xhol/T3
**Pax6**	AF 154552, positions 553 to 1074	Dr. Sylvie Rétaux, CNRS, Paris, France	SaII/T7

Riboprobes were synthesized in the presence of digoxigenin-11-UTP or fluorescein-12-UTP (Roche Diagnostics, Mannheim, Germany) for antisense and sense control probes. The embryos were processed *in toto* (see Bachy et al., 2001; Moreno et al., 2004). Hybridization step was conducted with 2 μl/ml of a DIG-labeled RNA probe, in a 50% formamide-containing medium, overnight at 55°C. The solution used for prehybridization (at 60°C for 2 h) and hybridization contained 50% deionized formamide, 5× standard saline citrate, 2% blocking reagent (Roche Diagnostics), 0.1% Tween-20, 0.5% 3-[(3-cholamidopropyl)-dimethylammonio]-1-propanesulfonate (CHAPS; Sigma-Aldrich, Steinheim, Germany), 1 mg/ml of yeast tRNA (Sigma-Aldrich), 5 mM of ethylenediaminetetraacetic acid (Sigma-Aldrich), and 50 μg/ml of heparin (Sigma-Aldrich) in water. Hybridization was detected using an alkaline phosphatase-coupled anti-DIG antibody (Roche Diagnostics, dilution 1:1, 500). Alkaline phosphatase (AP) staining was developed in some of them with 4-nitroblue tetrazolium chloride/phosphate/ 5-bromo-4-chloro-3-indolyl-phosphate solution (NBT/BCIP) and in others, with fast red tablets (Roche Diagnostics). After hybridization, the brains of the embryos reacted *in toto* were immersed in a solution of 30% sucrose in PB for 4–6 h at 4°C. They were then embedded in a solution of 30% sucrose and 20% gelatin in PB and stored overnight at 4°C in a solution of 3, 7% formaldehyde and 30% sucrose in PB. Sections were cut at 10–16 μm thickness in classical transverse, horizontal or sagittal plane on a freezing microtome. A set of brains were not cut and were processed for IHC in toto, as described below.

### Double ISH

Selected embryos were fixed and processed in toto, for ISH with digoxigenin-UTP-labeled antisense riboprobes (Gbx2, Xiro1, Tcf4 [Tcf7L2]), where each of those was combined with fluorescein-UTP-labeled antisense riboprobes of Shh in hybridization buffer at 55°C overnight. Shh was the first probe to reveal following the same steps that in simple ISH using an AP-coupled anti-fluorescein antibody (Roche Diagnostics, dilution 1:1,500), by NBT/BCIP and was observed in blue color. After that, the embryos were treated with Glycine 0.1M-HCl-0.1%Tween (pH 2.2) to break the AP unions, and were incubated with an AP-coupled anti-DIG antibody (Roche Diagnostics, dilution 1:1,500). AP staining was developed with INT (2-[4-iodophenyl]-3-[4-nitrophenyl]-5-phenyltetrazolium chloride)/BCIP solution (Roche Diagnostics, Mannheim, Germany) and was observed in orange color.

### Immunohistochemistry (IHC)

Different two-step protocols for bright field or immunohistofluorescence (IHF) were conducted after *ISH*. Antibody cocktails were used as follows: (1) Incubation at 4°C for 72 h in a mixture of mouse anti-Pax7, mouse anti-Nkx2.2 or mouse anti-Isl1 (Developmental Studies Hybridoma Bank, Iowa City, IA, USA; diluted 1:500 in PB containing 0.5−1% Triton X-100 [PB-T]) (Table 3) after hybridization; (2) second incubation was for 90 min in biotinylated horse anti-mouse (1:100, Vector, Burlingame, CA, USA; catalog reference: BA-2000); and (3) the avidin-biotin complex (ABC) standard kit (Vector, Burlingame, CA, USA: SK4100) was used next as recommended by the manufacturer, with appropriate washing steps. In the case of immunohistofluorescence, second incubation was for 90 min in Alexa 488-conjugated goat anti-mouse (Molecular Probes; catalog reference A21042), followed by washing steps to finish the reaction. In some cases, single bright field IHC was performed for Nkx2.2 or Pax7 as above, without combination with ISH.

**Table 3:** **List of antibodies used in the present study**.

Antigen	Immunogen	Type; manufacturer; catalog No.	MW(kDa)	Dilution
PAX7	Recombinant protein made in *E.coli*, containing the amino acids 352-523 of chicken PAX7 protein	Monoclonal mouse anti-PAX7; Developmental Studies Hybridoma Bank; catalog reference: Pax7-c	55	1:500
GABA	GABA-BSA	Polyclonal rabbit anti-c-minobutyric acid. Sigma; catalog reference: A2052	0.0103	1:3000
Nkx2.2	*E.coli*-derived recombinant chick NKX2.2 NK2 factor related	Monoclonal mouse anti-Nkx2.2; Developmental Studies Hybridoma Bank; catalog reference: 74.5A5	30	1:500
Islet1	Amino acids 247-349 at the c-terminus of rat Islet1	Monoclonal mouse-anti-Isl; Developmental Studies Hybridoma Bank; catalog reference: 40.2D6	39	1:500

### Double Immunohistofluorescence

Immunohistofluorescence procedures were carried out *in toto* embryos as follows: (1) First incubation was conducted for 72 h at 4°C in the dilution of a mixture of primary antibodies (Table 3), combining mouse anti-Nkx2.2 with rabbit anti-GABA; and (2) Second incubations were conducted with Alexa 594-conjugated goat anti-rabbit (Molecular Probes, Eugene, OR; catalog reference A11037), and Alexa 488-conjugated goat anti-mouse (Molecular Probes; catalog reference A21042). Both secondary antibodies were diluted 1:500 in PB containing 0.5–1% Triton X-100, and incubation lasted 90 min at room temperature. Finally, the embryos were gelatin blocked (as detailed above) and cut on a freezing microtome at 14 μm in the transverse, horizontal, or sagittal plane. After being rinsed, the sections were mounted on glass slides and coverslipped with Vectashield mounting medium (Vector Laboratories, Burlingame, CA, USA; cat. no. H1000).

### Specificity and Characterization of the Antibodies

The antibodies used in this study (Table 3) have been previously tested in *Xenopus*; many of them were used as territory markers in the brain, and the patterns of staining showed the same distribution as that observed in the present study (Moreno et al., 2008a,b; Morona and González, 2008; González and Northcutt, 2009; Domínguez et al., 2011; Morona et al., 2011; Bandín et al., 2013). Controls for the immunohistochemical procedures included: (1) Western blot analysis; (2) incubation of some selected sections with preimmune mouse or rabbit sera instead of the primary antibody; (3) controls in which either the primary or the secondary antibody was omitted; and (4) predsorption of the primary antibodies with synthetic peptides.

The specificity of the antibodies used has been assessed by the commercial suppliers (Table 3); in addition, immunoblotting was conducted. The Western blots of brain extracts of *Xenopus laevis* showed that all antibodies used labeled a single band, which with small variations corresponded well with the bands labeled in the rat lanes.

Western blot analysis with the Pax7 antibody detected a single band at the same molecular weight as the major product detected in rat brain extract (about 55 kDa) (Morona et al., 2011). This band most likely corresponds to the Pax7 protein in *Xenopus*, because it coincides with the calculated molecular weight in relation to the published nucleotide sequence for *Xenopus* Pax7 (NCBI accession number NM_001095526.1).

The anti-Nkx2.2 monoclonal antibody was developed by Dr. Jessell (Columbia University; New York). The DNA region of NK2 transcription factor of chick was cloned by polymerase chain reaction into the *E. coli* expression vector. Recombinant protein was expressed and purified. The monoclonal antibody was generated by immunization of mice with the recombinant protein. It has been tested in mice, rat, chick and human (see Developmental Studies Hybridoma Bank Data Sheet). The specificity of the Nkx2.2 antibody has been confirmed by an absence of labeling in Nkx2.2^−/−^ mice (Cai et al., 2010). Western blot analysis with the anti-Nkx2.2 antibody detected a single band at the same molecular weight as that of the major product detected in rat brain extract. This same antibody has also been tested by Western blot with chicken brain extracts and two bands were obtained of 43 kDa and 28 kDa and two isoforms were suggested (Ferran et al., 2009). The band observed in *Xenopus laevis* (Domínguez et al., 2013) corresponds well to the band of 28 kDa, observed in the chicken, the urodele amphibian *Pleurodeles waltl* (Joven et al., 2013a) and the turtle *Pseudemys scripta* (Moreno et al., 2012a).

The anti-GABA antibody was developed in rabbit using GABA-BSA as immunogen (Sigma; catalog reference A2052). The antibody was isolated from antiserum by immunospecific methods of purification, and antigen-specific affinity isolation removed essentially all rabbit serum proteins, including immunoglobulins that do not specifically bind to GABA. In previous studies, the same antibody has been used in different species, including *Xenopus laevis* (Bachy and Rétaux, 2006; Moreno and González, 2007; Moreno et al., 2008a) with comparable successful results, supporting its conserved specificity and cross-reactivity in all species studied. Furthermore the GABA staining obtaining with this antibody fully matches the distribution of GAD67 in *Xenopus* (Brox et al., 2003).

### Imaging

The labeled sections were analyzed with and Olympus BX51 microscope equipped for fluorescence with appropriate filter combinations. Selected sections were photographed using a digital camera (Olympus DP70). Contrast and brightness of the photomicrographs were adjusted in Adobe PhotoShop CS4 (Adobe Systems, San Jose, CA, USA) and figures were mounted in Canvas 11 (ACS Systems International).

## Results

**Figure 1:**
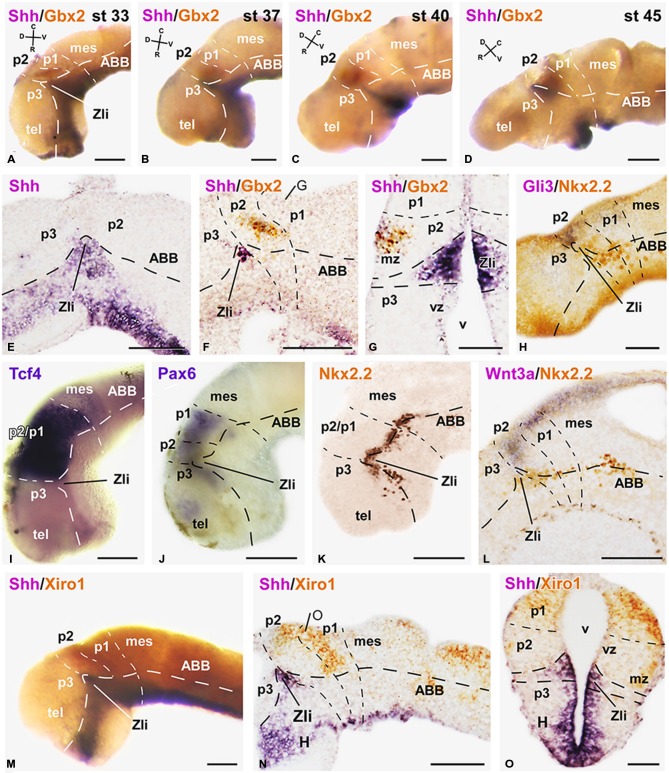
**Expression of early markers of the diencephalic prepatterning and formation of the Zli**. Microphotographs of lateral views of the forebrain in whole mounts labeled for double *in situ* hybridization (ISH) to reveal Shh (purple) and Gbx2 (orange) at the embryonic stages indicated **(A–D)**; the orientation is indicated in each panel to highlight the dorsoventral and rostrocaudal change of the axis through the thalamus at each stage. **(E–O)**: Microphotographs of whole mounts **(I,J,M)** and sagittal **(E,F,H,K,L,N)** or transverse **(G,O)** sections of embryos at stages 33/34. The photographs correspond to single ISH **(E,I,J)**, single immunohistochemistry (IHC; **K**), and combinations of double ISH **(F,G,M–O)** or combined ISH and IHC **(H,L)**. The markers labeled are indicated in the upper left of each photograph. All images are oriented following the same standard: dorsal is upwards in transverse and sagittal sections, and rostral is to the left in sagittal sections. The neuromeric boundaries and main brain subdivisions are indicated to assist in the precise localization of the labeling. The levels of the transverse sections **(G,O)** are indicated in photographs **(F,N)**, respectively. Scale bars = 100 μm **(E,F,L–N)**, 50 μm **(A–D,G–K,O)**. See list for abbreviations.

**Figure 2:**
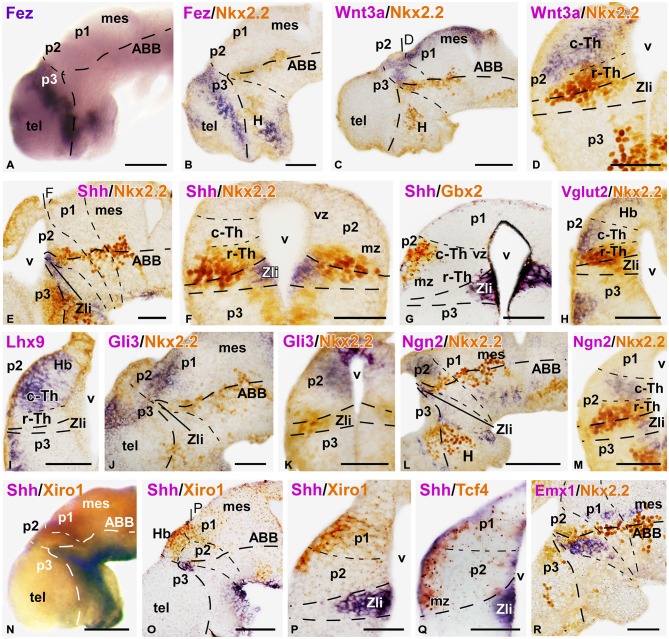
**Expression of thalamic markers at early embryonic stages 37/38**. Microphotographs of whole mounts **(A,N)** and sagittal **(B,C,E,J,L,O,R)** or transverse **(D,F–I,K,M,P,Q)** sections of embryos at stages 37/38. Photographs correspond to single ISH (purple; **A,I)**, double ISH (purple/orange; **G,N–Q)** and combination of ISH (purple) with IHC (brown) (**B–E**, **F,H,J,K,L,M,R**). The markers labeled are indicated in the upper left of each photograph. All images are oriented following the same standard: dorsal is upwards in transverse and sagittal sections, and rostral is to the left in sagittal sections. The neuromeric boundaries and main brain subdivisions are indicated to assist in the precise localization of the labeling. At these stage c-Th and r-Th subdivision of the thalamus were distinguished **(M)**. The levels of the transverse sections **(D,F,P)** are indicated in photographs **(C,E,O)**, respectively. Scale bars = 100 μm **(A,B,L,N,O)**, 50 μm **(C–K,M,P–R)**. See list for abbreviations.

**Figure 3:**
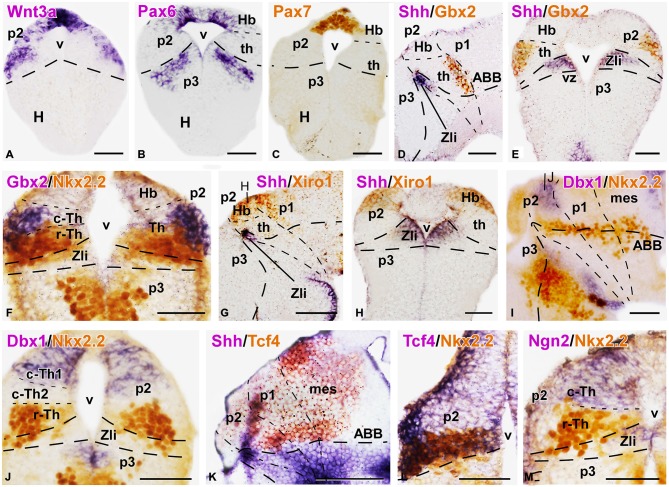
**Expression of thalamic markers at embryonic stages 40/41**. Microphotographs of transverse **(A,B,C,E,F,H,J,L,M)** or sagittal **(D,G,I,K)** sections of embryos at stages 40/41. Photographs correspond to single ISH (purple; **A,B)**, single IHC **(C)** double ISH (purple/orange; **D,E,G,H,K**) and combination of ISH (purple) with IHC (brown) **(F,I,J,L,M)**. The markers labeled are indicated in the upper left of each photograph. All images are oriented following the same standard: dorsal is upwards in transverse and sagittal sections, and rostral is to the left in sagittal sections. The neuromeric boundaries and main brain subdivisions are indicated to assist in the precise localization of the labeling. Note the mutually exclusive expression between Gbx2 in the thalamus and the Xiro1 in the habenular region, (compare **E** and **H**; **D** and **G)**. The levels of the transverse sections **(H,J)** are indicated in photographs **(G,I)**, respectively. Scale bars = 100 μm **(G,K)**, 50 μm **(A–F,H,I,K–M)**. See list for abbreviations.

**Figure 4:**
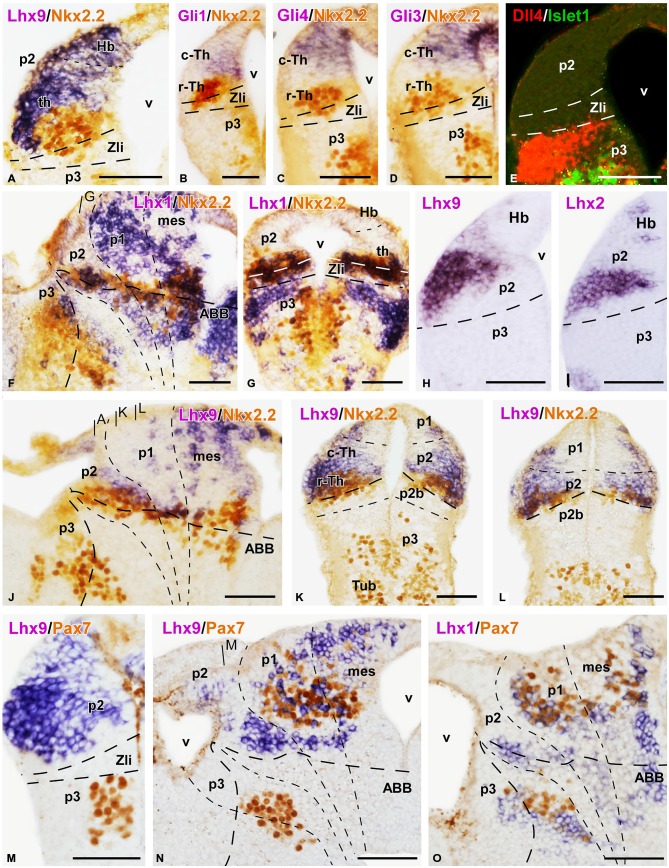
**Expression of thalamic markers at embryonic stages 40/41**. Microphotographs of transverse **(A–E,G–I,K–M)** or sagittal **(F,J,N,O)** sections of embryos at stages 40/41. In all cases, photographs correspond to combination of ISH (purple) with IHC (brown), except for a double fluorescent ISH (red) and IHC (green) panel **(E)** and two single ISH (purple; **H,I)**. The markers labeled are indicated in the upper left of each photograph. All images are oriented following the same standard: dorsal is upwards in transverse and sagittal sections, and rostral is to the left in sagittal sections. The neuromeric boundaries and main brain subdivisions are indicated to assist in the precise localization of the labeling. The levels of the transverse sections **(A,K,L)** are indicated in photograph **(J)**, the level of **(G)** is indicated in **(F)**, and the level of **(M)** is indicated in **(N)**. Scale bars = 50 μm. See list for abbreviations.

**Figure 5:**
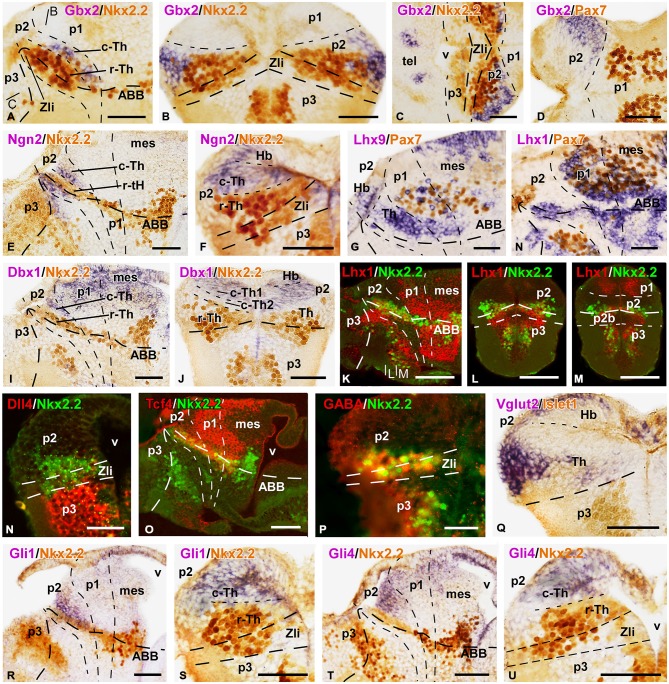
**Expression of thalamic markers at embryonic stage 45**. Microphotographs of sagittal **(A,E,G–I,K,O,R,T)**, transverse **(B,F,J,L–N,P,Q,S,U)**, and horizontal **(C,D)** sections of embryos at stage 45. In all cases, photographs correspond to combination of ISH (purple) with IHC (brown), except for the double fluorescent ISH (red) and IHC (green) panels **(K–O)** and a double fluorescent IHC **(P)**. The markers labeled are indicated in the upper left of each photograph. All images are oriented following the same standard: dorsal is upwards in transverse and sagittal sections, and rostral is to the left in sagittal sections; in the horizontal sections rostral is to the left. The neuromeric boundaries and main brain subdivisions are indicated to assist in the precise localization of the labeling. The levels of the transverse section **(B)** is indicated in photograph **(A)**, and the levels of **(L,M)** are indicated in **(K)**. Scale bars = 100 μm **(K–M,O)**, 50 μm **(A–J,N,P–U)**. See list for abbreviations.

**Figure 6:**
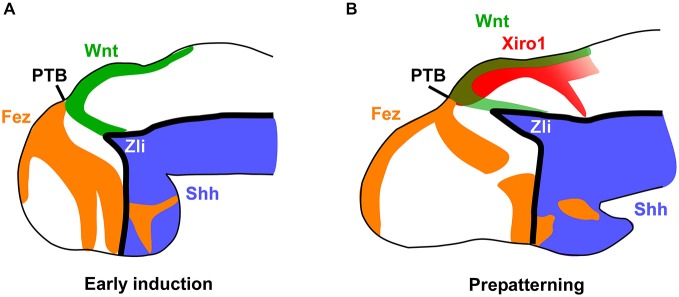
**Main markers involved in the early thalamic prepatterning**. Schemes of lateral views of the forebrain at early and late embryonic stages representing the main expressed factors that lead in *Xenopus* to a first induction of the thalamic region and the position of the Zli **(A)**, and the subsequent prepatterning of the thalamus and formation of the Zli **(B)**. The prethalamo-thalamic boundary (PTB) can be inferred in the zone where the rostral and caudal expressions abut.

**Figure 7:**
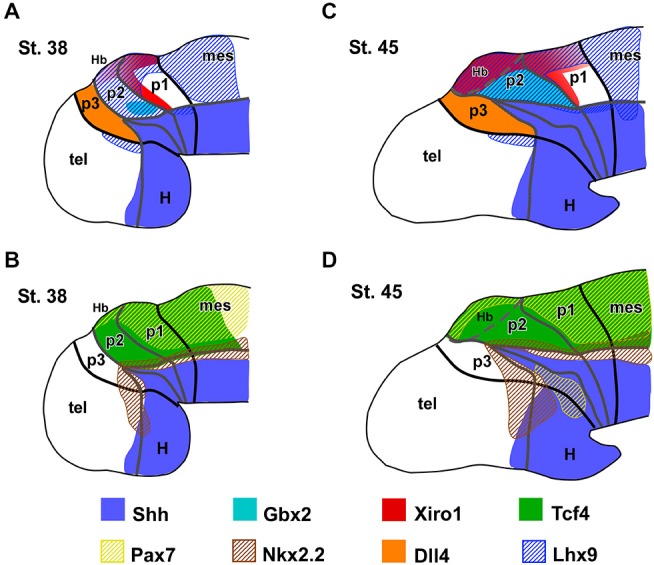
**Expression patterns of the main thalamic markers**. Summary diagrams representing in lateral view of the forebrain the extent of the expressions of the main markers analyzed in *Xenopus* with respect to the thalamus and neighboring regions at early **(A,B)** and late **(C,D)** embryonic stages.

**Figure 8:**
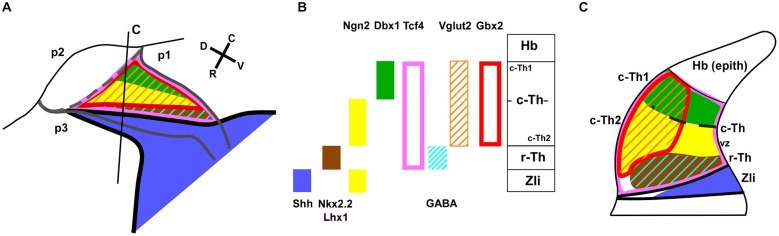
**Diagram of the main expression patterns for markers of the thalamic subdivisions**. Schematic representation of the early progenitor domains in the thalamus of *Xenopus*, indicating the main markers found in each domain in the present study. **(A)** is a drawing of a lateral view of the thalamus in which a color-code for the distinct expression patterns for the markers used is depicted in **(B)**. **(C)** corresponds to the representation of a transverse section at the level indicated in **(A)** in which the extent of the thalamic progenitor domains is indicated in relation to the Zli.

To describe the molecular patterns that progressively characterize the thalamic territory as embryonic development proceeds, we will present results obtained in whole mount specimens and in series of transverse, sagittal, and horizontal sections. We performed two-color ISH or IHC, and combinations of ISH and IHC to identify landmarks (like the Zli) and obtain precise spatial gene expression maps in the developing thalamus and adjacent regions. We analyzed the embryonic development starting at stage 29/30, when [3H]-thymidine studies indicated that in *Xenopus* distinct parts of the diencephalon, including the epithalamus, arise (Tay and Straznicky, 1982). From these early stages until stage 40, in which neuron generation appears in the thalamus (Zeng et al., 2008), the prepatterning of the thalamus and the formation of the Zli take place. Figures 1 and 2 illustrate the expression patterns observed at early stages. During the late embryonic stages (stages 40–45) the patterning of the thalamus is established, and the features of the different gene expression patterns are shown in Figures 3–5. Schematic representations of the main expression patterns occurring during prepatterning (Figure 6) and the different patterns observed along the embryonic period are drawn in a lateral view of the forebrain (Figure 7). In addition, we will comment on the expression observed in cells in or close to the ventricular lining (ventricular zone; vz) and in migrated cells in the external zones (mantle zone; mz). A summary diagram of the combinatorial gene code found in each thalamic region is provided (Figure 8).

### Prepatterning of the Embryonic Thalamus (Stages 30–36)

Along the developmental period covered in our study (stages 29–45) the brain of *Xenopus* embryos changes in morphology as the longitudinal axis is straightened, and the progressive differences in the diencephalic organization can be inferred by the labeling of two main markers, Shh and Gbx2 (Figures 1A–D). Thus, the two-color ISH used showed that the Shh expression labeled the developing Zli, highlighting the localization of the thalamus, just caudal to it, and roughly labeled for Gbx2.

In early embryos, the expression of Shh was observed predominantly in the ventral neural tube but, at levels of the diencephalon, the Shh expression extended dorsally in the basal part of the forming Zli (Figures 1A,E). The extent of the Shh expression in the Zli at stages 31–34 (about 40 h postfertilization) reached halfway dorsally into the alar plate, progressively forming a wedge-shaped zone where the Shh expression was restricted to the medial region, close to the ventricle (Figures 1A,B,E–G,M–O). It subsequently appeared as a prominent wide spike that progressed dorsally as a narrow strip dividing most of the alar territories of p3 and p2, which will develop into the prethalamus and thalamus, respectively.

In the diencephalic alar plate of embryos before stage 33, Pax6 expression occupied the alar domains, sharply highlighting the p1-mesencephalic boundary (Figure 1J). Subsequently, as the Shh expression of the Zli progressed dorsally, the Pax6 labeling diminished caudally to it, i. e. in the thalamus, whereas it persisted in the prethalamus (data not shown, see Moreno et al., 2008b). In addition, strictly dorsal to the extending Shh expression in the Zli, a small wedge-shaped patch of Gli3 expression was noted in early embryos (Figure 1H), which expanded into the thalamus at latter stages (see Figures 2J,K).

Interestingly, Wnt3a expression in the dorsal part of the developing thalamus was also noted at early stages (Figure 1L) and its rostral expression domain was abutting the Zli, whereas caudally it was also expressed in the dorsal p1 and mesencephalon. At later stages, the Wnt3a expression was more widely distributed in the caudal part of the thalamus (Figures 2C,D).

Other markers of the thalamic region during early embryonic stages included the transcription factor Tcf4, whose expression extended from the p3/p2 boundary (marked by the Zli) to the mesencephalon (Figure 1I). At stages 37/38 (about 60 h postfertilization), Tcf4 expression was continued in p1 and p2, predominantly in the mz (Figure 2Q). The Iroquois-related homeobox gene Xiro1 was also expressed caudal to the Zli in the posterior diencephalon and continued in the midbrain and hindbrain (Figure 1M). In p1, the expression showed a rostrocaudal gradient and the intense reaction in the rostral part highlighted the caudal boundary of p2 (Figure 1N). Dorsally in p2, the expression of this marker showed a sharp anterior border at the level where the Zli was growing, and the expression was observed at the lateral aspect of the mz of p2, whereas it labeled the vz and mz of p1 (Figures 1N,O). At slightly later stages the embryos showed Xiro1 expression more restricted in p1, while in p2 the expression was intense in the dorsal epithalamus and only low levels of expression were observed in the caudal and lateral mz of the thalamus (Figures 2N–P).

The rostral boundary of the embryonic thalamus at the dorsal part of the brain was also assessed by the expression of Fez (also known as Fezl1), which strongly labeled the dorsalmost part of p3 in contrast to the total lack of expression in p2, thus highlighting the p3/p2 boundary (Figures 2A,B). Therefore, the expression domain of Fez abutted dorsally the anterior border of Xiro1 (Figures 2A,N) and Wnt3a (Figures 2B,C).

Starting by stage 30, the homeobox gene Nkx2.2 was expressed adjacent to the Shh expresión domain, forming a narrow band along the neural tube and in the neural epithelium flanking the developing Zli. The progressive dorsalward ascent of the Shh expression in the Zli was accompanied by a corresponding progressive modification of the longitudinal band that expressed Nkx2.2, which was pushed upward by the Zli as it formed (Figures 1H,K,L).

### Initial Recognition of Rostral and Caudal Thalamic Domains (Stages 37–38)

The embryonic thalamus early formed two molecularly distinct subdivisions: the small rostral thalamus (r-Th) showing the expression of Nkx2.2, and the larger caudal thalamus (c-Th) that expressed Gbx2, Ngn2 and Lhx9, among others, already at early stages.

By stage 32, Nkx2.2 expression was observed flanking the Zli, marked by Shh, and this expression gradually increased throughout the embryonic development (Figures 2D–F,H,M,R). Shh expression was located in the vz of the Zli, whereas Nkx2.2 expression was detected in the vz and mz of the rostral p2 and in the vz in the caudal p3, without overlapping the Shh expression zone (Figures 2E,F). Thus, the rostral part of the thalamus that is immediately adjacent to the Zli and expressed Nkx2.2 was recognized as the r-Th in *Xenopus* from these early stages. Other markers, such as Emx1, were clearly located in basal part of p2 (p2b) partially overlapping the Nkx2.2 domain, and continued caudally in the alar p1 and mesencephalon (Figure 2R).

The c-Th is a large territory caudal to the r-Th and rostral to the alar territory of p1 (pretectal region). Dorsal to the c-Th develops the epithalamus (habenular and pineal regions) that is molecularly distinct. Expression of Gbx2, which was located in postmitotic cells in the mz in a small region close to the basal boundary in the early thalamus (Figures 1F,G), extended later in the mz of the c-Th with the highest levels of expression in the superficial zone, far from the expression zone of Shh (Figure 2G). However, the Gbx2 expression did not extend dorsally into the epithalamus and caudally abutted the pretectum (Figure 1B), highlighting the thalamic boundaries. Differently, Wnt3a expression was found more extensively in the c-Th, in both the mz and, to a lesser extent, in the vz. However, the most superficial and periventricular zones showed low Wnt3 expression (Figure 2D). The expression of Wnt3a abutted the Fez expression at the dorsal part of the Zli (compare in Figures 2A–C) and, in addition, was expressed in the epithalamus and extended caudally in the dorsal p1 and mesencephalon (Figure 2C).

At earlier stages, Gli3 expression occupied a narrow zone above the Zli and it was seen later in the dorsal aspect of the c-Th and pretectum, both in the vz and mz (Figures 2J,K), whereas, Xiro1 expression was mainly confined to the superficial mz in p2 (Figure 2P). Tcf4 also labeled intensely the superficial part of the mz throughout p2 and continued in p1 (Figure 2Q).

By stage 37, Ngn2 expression was detectable in the vz at the Zli and in the vz and deep part of the mz of the c-Th (Figures 2L,M), in contrast to the Lhx9 expression, which became recognizable mainly in the superficial mz (Figure 2I). Also at the superficial part of the mz of the c-Th and the epithalamus, Vglut2 (a vesicular glutamate transporter) expression was observed for the first time at these stages (Figure 2H).

### Patterning of the Thalamus at the End of the Embryonic Period (Stages 40–41)

*Xenopus* embryos at stage 40 (about 70 h postfertilization) are characterized by the opening of the mouth and strikingly increase of body length (6.3–6.8 mm). The morphology of the forebrain also changes notably from previous stages and a fast progressive enlargement of the secondary prosencephalon (hypothalamus and telencephalon) takes place (Figure 1C). In the diencephalic alar plate, the thalamic territory increased markedly in size and the Zli showed a spike domain of Shh expression, thinner than in early embryos, medially restricted to the neuroepithelium (Figures 1C and 3D,E,H).

During these stages, Wnt3a expression in p2 was located in the c-Th and the dorsal epithalamus (Figure 3A). In the c-Th the expression was mainly found in the mz, whereas in the epithalamus (mostly at the dorsal midline region) strong Wnt3a expression was observed in the vz. The pattern of Pax6 expression also changed strikingly (Figure 3B) and most of the broadly expressing thalamic territory did not longer show Pax6 labeling at these stages, whereas it persisted in the alar part of p3 and p1. Within p2, Pax6 expression remained only in the dorsal region that will give rise to the epithalamic formation (Figure 3B). Of note, expression of another member of the Pax gene family, Pax7, was found only in the dorsal part of p2 and continued caudally into the alar p1 (Figure 3C).

The division of the thalamic progenitor regions into r-Th and c-Th domains at stage 40 was discernable by labeling combinations for Nkx2.2, specific of r-Th, and the set of markers identified for the c-Th in previous stages (Figures 3D–M, 4). The region expressing Nkx2.2 from the vz to the mz increased laterally in the r-Th, and the intensity of the IHC was higher in the mz (Figure 3F). Gbx2 was distinctly expressed in the mz of the c-Th, and the boundary between the c-Th and the epithalamus (dorsally) and the alar p1 (caudally) was sharply defined (Figures 3D–F). Moreover, double labeling for Nkx2.2 and Gbx2 revealed that cells expressing each marker overlapped in the superficial mz (Figure 3F).

Other markers of the c-Th division included Xiro1 (Figures 3G,H) and Tcf4 (Figures 3K,L). In contrast to Gbx2, both Xiro1 and Tcf4 were expressed in the developing habenula, in the dorsal part of p2. Xiro1 expression in the dorsal p2 (epithalamus) was intense (Figure 3H) and it marked the boundary with the alar p1, where it showed a gradient of expression decreasing from the rostral part (close to p2) toward caudal pretectal regions (Figure 3G). The pattern of labeling for Tcf4 was different, extending in the epithalamus and c-Th, but also in the r-Th where it overlapped the Nkx2.2 expressing zone (Figure 3L), above he Shh expression of the Zli (Figure 3K). Comparatively, Lhx9 at these stages showed a pattern of expression similar to that of Tcf4, labeling primarily mz cells in the c-Th and extending dorsally (Figure 4A).

Interestingly, the comparison of the expression pattern of Ngn2 and Dbx1 in the doubly labeled sections allowed a further division of the c-Th into caudodorsal c-Th1 and rostroventral c-Th2 parts. Thus, Ngn2 expression highlighted the extent of the whole c-Th (Figure 3M), whereas Dbx1 was restricted to the c-Th1 region, leaving the c-Th2 region free of expression (Figures 3I,J). The pattern of the Gli factors (Gli1, Gli3, and Gli4) also showed distinct distribution in the thalamus at these stages (Figures 4B–D). Gli1 expression was found mainly in the rostral part of the c-Th, adjacent and partly overlapping the Nkx2.2 positive r-Th, primarily in the vz and deep mz (Figure 4B). In turn, Gli3 and Gli4 were expressed more caudodorsally (coinciding with the c-Th1), including the epithalamus (Figures 4C,D).

The analysis of the different LIM-homeodomain genes in the developing diencephalon was also useful to characterize the distinct thalamic subregions. Some markers of the LIM family, such as Islet1 were previously shown to label neighboring regions of p2 that, together with other markers (like Dll4) served to highlight the thalamic boundaries (Figure 4E). However, more information was obtained analyzing the pair Lhx1/Lhx5, which was expressed in the r-Th and the pretectum (Figures 4F,G), and the pair Lhx2/Lhx9 that was primarily confined to the c-Th and, to a lesser extent the epithalamus (Figures 4H,I). In particular, the combined analysis of the expressions of Lhx9 and Nkx2.2 in the same sections (Figures 4J–L) showed that in the ventrolateral part of the thalamus the expression of Lhx9 overlapped that of Nkx2.2, starting in the lateral thalamic region at mid dorsoventral levels (Figures 4J,K) and expanding ventrally (Figure 4L). Finally, the analysis of the expression for Pax7 in combination with Lhx9 and Lhx1 served to interpret the wide distribution of the Lhx9 expression in the thalamus in contrast to the restricted Lhx1 expression limited to the r-Th, highlighted by the expression of Pax7 in the neighboring p3 and p1 (Figures 4M–O).

### End of the Embryonic Period (Stages 44–45)

This period starts at the time the embryos begin spontaneous swimming, and continues for about 3 days, when the yolk is completely absorbed. It ends when the animals initiate to feed independently, which marks the beginning of the long larval period (stage 45). During the late embryonic stages, the brain changed considerably, the Zli extended more dorsally, penetrating most of the alar territory between p2-p3. In addition, the alar part of p2, including the thalamus and the epithalamus, increased in size and complexity (Figures 1D and 5).

At these stages, the Nkx2.2 expressing region (including the r-Th) increased in size and the intensity of the immunoreaction was higher than at previous stages (Figures 5A–C,F). Markers of the c-Th, primarily Gbx2, Tcf4, Ngn2 and Lhx9 unraveled an enlargement of this region at the lateral aspect of the thalamus (Figures 5A–G,O). In addition, the expression profile of the epithalamus included Lhx9, Ngn2, Pax7, and Tcf4 (Figures 5F–H,O). Of note, Dbx1 expression continued to show a gradient of expression being more intense in the habenula and the c-Th1, with almost total lack of expression in c-Th2 (Figures 5I,J), where Ngn2 expression was located (Figures 5E,F).

In the region of the r-Th, labeled for Nkx2.2, overlapping expressions of Lhx1 (Figures 5K–M), Tcf4 (Figure 5O) and, only dorsomedially, Gli1 (Figure 5R) were observed. Already at these stages, numerous cells of the Nkx2.2 positive r-Th region were double labeled for GABA (Figure 5P). In turn, the region of the c-Th, and also the developing habenula, showed expression for Vglut2 (Figure 5Q) and Gli4 (Figures 5T,U). In line with the observations in previous developmental stages, the most intense Vglut2 expression was located in cells that occupied a wide superficial region of the mz in c-Th, and also in the superficial epithalamic region (Figure 5Q). In relation to the pattern of expression for Gli1 and Gli4 factors, whereas Gli1 labeled primarily the rostrodorsal part of the thalamus, even partially overlapping the Nkx2.2 expressing zone (Figures 5R,S), labeling for Gli4 was mainly located in the c-Th with a rostral (high) to caudal (low) gradient of expression and leaving free of labeling the lateral region where Gbx2 and Vglut2 were intensely expressed (Figures 5T,U).

Regarding the Gli transcription factors, Gli1 labeled primarily the rostrodorsal part of the thalamus, even partially overlapping the Nkx2.2 expressing zone (Figures 5R,S), whereas Gli4 was mainly located in the c-Th with a rostral (high) to caudal (low) gradient of expression, leaving free of labeling the lateral region where Gbx2 and Vglut2 were intensely expressed (Figures 5T,U).

## Discussion

In the present study, the developing thalamus of *Xenopus laevis* was examined morphologically by using combinations of markers revealed by ISH or IHC. In general, one of the markers in each combination corresponded to a well-characterized reference probe such as the Zli marker Shh (Domínguez et al., 2010), or a well-established immunohistochemical distribution for a transcription factor such as Nkx2.2 (Domínguez et al., 2011). Thus, we could unambiguously identify regional domains of expression for numerous genes in the thalamus, during embryonic development. In addition, the analysis of transverse, sagittal, and horizontal provided three-dimensional information, important to precisely annotate expression patterns in the complex diencephalon.

The early molecular profile of the thalamus has been described primarily in mouse and chicken embryos (reviewed in Puelles and Martínez, 2013). We have confirmed that orthologous genes with comparable expression patterns are found in the thalamus of *Xenopus*, highlighting molecular conservation between amniote and anamniote tetrapods. Furthermore, the spatiotemporal patterns of expression found in the thalamus of *Xenopus* for most markers analyzed confirmed also important shared features among vertebrates. Bellow we discuss our data in relation to main events in thalamic early development, attending to the acquisition of thalamic competence in the prospective alar part of the p2 diencephalic segment (prepatterning), the formation of the Zli, and the early specification of thalamic domains (patterning). All the data will be interpreted according to the current prosomeric model (Puelles and Rubenstein, 2003).

### Prepatterning of Prospective Thalamus and Formation of the Zli

Fate-mapping analysis in combination with studies of lineage restriction have unraveled the morphological and molecular characteristics of the thalamus (Figdor and Stern, 1993; Larsen et al., 2001; García-López et al., 2004). Numerous genes showed early expression patterns that varied significantly in relation to the developing forebrain subdivisions (Bell et al., 2001; Sánchez-Arronés et al., 2009). However, groups of genes become eventually expressed in distinctly in the thalamus (Puelles and Martínez, 2013). Thus, the formation of distinct identities in the developing brain is the result of the combinatorial expression of sets of transcription factors that form the so-called prepattern, which characterize at these stages and ulteriorly the tissular differentiation and provide corresponding positional references that serve to define the identity of particular regions in the CNS (reviewed in McGinnis and Krumlauf, 1992; Rubenstein et al., 1994; Gómez-Skarmeta et al., 2003).

The same mechanisms described to act in the spinal cord mediate *dorsoventral* regionalization in the diencephalon, including the thalamic region (Hashimoto-Torii et al., 2003; Jeong et al., 2011). Thus, Shh is expressed along the longitudinal axis in the floor and basal plates of the diencephalon, mesencephalon and rhombencephalon. In particular, Shh in p2 is early expressed in the floor and basal plates and in the underlying notochord, whereas BMPs and Wnts appear expressed at the roof plate (Hollyday et al., 1995; Zhang et al., 2014). The same signaling events are likely at early thalamic development of *Xenopus*, strongly suggested by the expression observed for Shh and Wnt (present results; Domínguez et al., 2010).

In turn, for the *anteroposterior* diencephalic regionalization several distinct expression mechanisms have been described that account for the early thalamic prepatterning. It is generally accepted that rostral vs. caudal forebrain differentiation depends on rostral inhibition of Wnt signaling, whereas Wnts remain active in the prospective diencephalon and midbrain (Niehrs, 1999; Kiecker and Niehrs, 2001; Houart et al., 2002). Wnt signals possibly cause the caudal downregulation of Six3, as well as the induction of Irx3 expression caudal to Six3, in the region fated to become caudal diencephalon (Braun et al., 2003). In the early chick embryo, it was proposed that the site of the Zli formation and the rostral thalamic boundary are marked by the abutting expression zones of Six3 rostrally and Irx3 caudally, both of which seem to be involved in mediating differential competence at pre-Zli stages (Kobayashi et al., 2002; Braun et al., 2003; Echevarría et al., 2003; Kiecker and Lumsden, 2004; Vieira et al., 2005). However, in other vertebrates a gap was observed between the Six3 expressing zone and those of orthologous Irx genes, such as Irx1 in mouse (Hirata et al., 2006), and both Irx1b and Irx7 in zebrafish (Lecaudey et al., 2005). Moreover, Six3-deficient mice show severe diminution of the region rostral to the Zli, but do express Shh in the Zli and posses rudimentary rostral tissue (Lagutin et al., 2003). This situation would suggest that other genes may cooperate with Six3 or may function parallel to it to specify the position of the Zli and regulate the formation of the rostral diencephalic region (Wilson and Houart, 2004; Kiecker and Lumsden, 2005). Subsequent findings in fish, chicken, and mouse pointed to the role of the forebrain embryonic zinc-finger genes Fez (Fezf1 and Fezf2), which are additional early genes expressed in the telencephalon and rostral diencephalon (Hashimoto et al., 2000; Matsuo-Takasaki et al., 2000; Hirata et al., 2006; Jeong et al., 2007), and were implicated in the rostrocaudal patterning of the diencephalon and in the establishment of the Zli by interaction with Irx and Wnt located in the dorsal neural tissue caudal to the prospective Zli. In particular, Wnt signaling was suggested to be involved in the formation of the forebrain rostrocaudal polarity regulating the expressions of Six3 and Irx (Itoh et al., 2002; Lim and Golden, 2007).

Comparatively, our detailed analysis in the amphibian *Xenopus laevis* demonstrates the early expression of Fez in the developing telencephalon and the rostral diencephalon almost abutting dorsally the region expressing Wnt3a and Xiro1 (orthologous to the Iroquois genes Irx1 in mouse, Irx3 in chick and Irx1b/ Irx7 in zebrafish; Gómez-Skarmeta et al., 1998) in p2 (Figure 6). The small gap between their expressions is gradually occupied by the growing Zli. These obsevations give us an idea of a high degree of evolutionary conservation of the interaction between these expressions to define the rostral boundary of p2 and the positioning of the Zli. In other vertebrates, Fez and Irx genes delineate the borders of the Zli through mutual repressive functions (Zeltser, 2005; Hirata et al., 2006; Scholpp and Lumsden, 2010), and similar mechanisms are likely in amphibians between Fez and Xiro1, probably in combination with Otx1/2 and other repressors expressed more rostrally (Rodríguez-Seguel et al., 2009).

Other two markers expressed in the early thalamic region of *Xenopus* with importance in the prepatterning are Tcf4 and Pax6. The transcriptional output of the Wnt signaling pathway is mediated by the Tcfs effectors (Arce et al., 2006; Archbold et al., 2012). Only Tcf7L2 is expressed in the developing mouse brain after E10.5 (Oosterwegel et al., 1993; Korinek et al., 1998). The expression domain of Tcf7L2 largely identifies the thalamus, pretectum and alar part of midbrain (Oosterwegel et al., 1993; Cho and Dressler, 1998; Jones and Rubenstein, 2004; Bluske et al., 2009). Comparatively in amphibians, among the Tcf transcription factors, only Tcf4 (equivalent to Tcf7L2) has been implicated in the anteroposterior patterning of the CNS (Koenig et al., 2010), and is expressed in the *Xenopus* embryo in the thalamic and pretectal regions, showing a distinct rostral boundary at the Zli (present results, Morona et al., 2011; see Figures 7B,C and 8A,B). Pax6 is a general marker of the prosencephalic alar plate in the early neural tube and its contribution to early diencephalic prepatterning is fundamented by the observation that its mutation causes abnormalities in prethalamus, thalamus, and pretectum (Stoykova et al., 1996; Grindley et al., 1997; Warren and Price, 1997; Pratt et al., 2000; Manuel and Price, 2005). Furthermore, Pax6 was demonstrated to be required for the establishment of the anlage of the middiencephalic organizer in zebrafish (Chatterjee et al., 2014) and mice (Caballero et al., 2014). In the early *Xenopus* embryos Pax6 extends throughout the diencephalic alar plate (including the prospective habenula in the epithalamus) and, at very early developmental stages, the posterior limit of the Pax6 expression coincides with that of Tcf4 (present results, Morona et al., 2011).

The aforementioned restricted patterns of gene expression delimit the region of the Zli in which Shh is expressed (Figures 6–8). Shh released from the Zli regulates through a concentration gradient the regionalization and patterning of the thalamus in mice (Jeong et al., 2011), chick (Kiecker and Lumsden, 2004; Vieira et al., 2005), and zebrafish (Scholpp et al., 2006). The expression of this morphogen ascending dorsally from the basal plate allowed us the observation of the progressive development of the Zli, as it was seen in different vertebrates including lamprey (Osorio et al., 2005), zebrafish (Barth and Wilson, 1995; Scholpp et al., 2006), frog (Ruiz i Altaba, 1998), chick (Vieira et al., 2005), and mouse (Shimamura et al., 1995). The expression of Shh in the Zli gradually progresses from the basal plate (also expressing Shh) but it was demonstrated that induction of Shh in the Zli does not depend on ventral Shh signaling in the zebrafish (Scholpp et al., 2006). Several developmental genes are controlled by Shh, including itself, and define diencephalic regionalization and cell fate, both rostrally in the prethalamus and caudally in the thalamus (Kiecker and Lumsden, 2004; Vieira et al., 2005). Thus, during the Zli maturation, Shh occupies the free-region between Fez and Irx (Xiro1) expressions in *Xenopus laevis* (present results; Figure 6).

Current conception of the Zli considers it as a mid-diencephalic secondary organizer with morphogenetic activity along the antero-posterior axis that releases as secreted morphogens Wnt proteins (Wnt8b, Wnt3a) and essentially, Shh (reviews in Parr et al., 1993; Vieira et al., 2005; Scholpp and Lumsden, 2010; Martínez et al., 2012). Thus, the developing diencephalic alar regions are exposed to anteroposterior Shh signals from the Zli that complement ventrodorsal Shh signaling originated at the underlying floor and basal plates (Vieira and Martinez, 2006; Jeong et al., 2011). Interestingly, in chick embryos the Zli formation has been centered on the role of Wnt8b, whose mid-diencephalic alar expression clearly precedes Zli formation (Garda et al., 2002; García-López et al., 2004; Puelles and Martínez, 2013). Its initially wedge-shaped expression domain is progressively reduced to a narrow gap limited anteriorly by Fezf2 and Lrrn1 (prospective prethalamus) and posteriorly by Lfng, Irx1, Tcf7l2, and Wnt3 (prospective thalamus). At this transverse linear locus, the earlier general dorsal expression of Gli3 results weakened, just as the ascending Shh-positive Zli starts. In contrast to this observation, Wnt8b is expressed in *Xenopus* only early in development (Merzdorf and Sive, 2006) and the expression of Gli3 was observed dorsal to the region where the Zli is subsequently formed. Gli3 is regulated by Shh and acts as transcriptional repressor (Schimmang et al., 1992) mediating Shh signaling (Persson et al., 2002; Abbasi et al., 2010).

In summary, in *Xenopus* the positioning of the Zli starts early with a prepatterned diencephalon in which anteroposterior interactions are established, including those suggested by the differential expression of Pax6, Fez, Tcf4, Xiro1, Wnt3a, and Gli3 (Figure 7). These first characterize distinctly the molecular identities of anterior, intermediate, and caudal parts of the diencephalon (p3, p2, and p1). The prepatterning process includes the induction of a broad mid-diencephalic Gli3 expression domain (similar to the Wnt8b wedge-shaped domain in the chicken). This domain would fix the precise subsequent position of the Zli between the Fez-positive rostral diencephalic region and the Xiro1-positive intermediate diencephalic region. These transcription factors would participate in a network that leads to define the competent domain under the induction of Shh in the future mid-diencephalic organizer in the Zli (Scholpp et al., 2006). Of note, the observed progression of Shh dorsalward in the Zli does not correspond to ventral-to dorsal cell movement since the phenomena involved are entirely of an inductive nature acting upon primarily alar tissue (García-López et al., 2004; Zeltser, 2005; Guinazu et al., 2007; Scholpp and Lumsden, 2010) and neither the Shh secreted from the basal plate nor the underlying axial mesoderm are required for the formation of the Zli in the zebrafish (Scholpp et al., 2006).

### Patterning of the Thalamus: Recognition of Distinct Progenitor Domains

Patterning that precedes the formation of elaborate brain regions requires local organizers to establish concentration gradients of morphogenetic signal molecules to determinate cell position and fate in adjacent responsive tissues. Thus, in the thalamus the previous prepattern is modified by the functioning of the Zli at later neural tube stages, acting as a intradiencephalic anteroposterior signaling center or secondary organizer (Martínez, 2001; Echevarría et al., 2003; Vue et al., 2007; Scholpp and Lumsden, 2010; Jeong et al., 2011; Epstein, 2012; Hagemann and Scholpp, 2012).

It is currently accepted that Shh released from the organizer in the Zli regulates regionalization and patterning of the thalamus, as has been demonstrated elegantly in zebrafish (Scholpp et al., 2006), chick (Kiecker and Lumsden, 2004; Vieira et al., 2005), and mice (Jeong et al., 2011). Thus, Shh is important as external cue to control the expression of diverse transcription factors within the thalamus. A specific challenge has been to find a correlation between the expression patterns for many genes under the action Shh (and other signaling pathways) during the early stages of thalamic development and the nuclei and different neuronal subtypes formed postnatally (Vue et al., 2007, 2009; Suzuki-Hirano et al., 2011). The graded Shh signaling on the developing thalamus seems to determine the initial patterning of two discrete progenitor domains in which different concentrations of the morphogen lead to distinct expression of downstream genes (Hashimoto-Torii et al., 2003; Scholpp et al., 2006; Szabó et al., 2009; Vue et al., 2009; Epstein, 2012; Hagemann and Scholpp, 2012). These two domains in *Xenopus* have been termed here r-Th and c-Th, attending to its rostral and caudal topological location in the thalamus. High levels of Shh signaling are required just caudal to the Zli for the formation of the r-Th, which develops adjacent to the Zli and expresses Nkx2.2 (in mammals also Mash1, Sox14, Tal1 and Gad1; Scholpp and Lumsden, 2010). In turn, the c-Th, develops separated from the Zli under the influence of progressively lower levels of Shh that lead to subsequent expression of a variety of genes (Hashimoto-Torii et al., 2003; Vue et al., 2009), most of which have been demonstrated also in *Xenopus* in our study (e.g., Wnt3a, Gli, Gbx2, Ngn2, Dbx1, and Lhx9).

### Rostral Thalamic Domain (r-Th)

This progenitor domain is induced by the high levels of Shh released from the Zli and the ventral midline. This situation leads to the expression of the transcription factor Nkx2.2, which in *Xenopus* is observed from early embryonic stages. Actually, the Nkx2.2 expression precedes the formation of the Zli and is observed just above, and partially overlapping, the Shh expressing domain, in the basal plate along the midbrain and forebrain. This region has been recently termed the liminal band (see Puelles and Martínez, 2013). As we have seen in *Xenopus*, during the gradual dorsal extent of Shh in the forming Zli, the parallel zone of Nkx2.2 expression accompany this dorsal elevation and forms rostral (prethalamic) and caudal (thalamic) liminal domains, previously referred to as rostral and caudal shell domains, with respect to the Zli, reflecting precisely the situation reported for amniotes (Martínez and Puelles, 2000; Puelles and Rubenstein, 2003; Puelles et al., 2004). The r-Th domain corresponds to the narrow Nkx2.2 region formed just caudal to the Zli in the thalamus, i.e., the caudal liminal band that accompanies the Zli, and extends from a small ventricular zone to a wider region in the lateral mantle zone (see scheme in Figure 8). In mouse and chick, Shh signaling from both the Zli and basal plate are necessary for r-Th identity (Kiecker and Lumsden, 2004; Jeong et al., 2011). However, in zebrafish the lack of Shh expression in the ventral midline, but retaining in the Zli, led to normal thalamic development (Scholpp et al., 2006). It was suggested that this apparent species difference could be due to the earlier onset of Shh expression in the Zli of the zebrafish, compared to the mouse (Barth and Wilson, 1995; Epstein et al., 1999). In the mouse, the slower development and the high brain complexity achieved may respond to the use of the two sources of Shh in the thalamus to gain neuronal diversity, whereas zebrafish is based on only one source of Shh. Comparatively, in *Xenopus laevis* thalamic development is slower than in zebrafish and the genes downstream the graded Shh signaling show expression pattern in the thalamus similar to amniotes, suggesting common mechanisms in tetrapods.

The combination of Nkx2.2 with other markers like Gli1, Tcf4 and Lhx1 served to the identification of the r-Th domain in *Xenopus* (Figures 7, 8). The Shh effects are mediated by the the Gli genes, that are differentially expressed in thalamic progenitor cells (Hashimoto-Torii et al., 2003). Our results show that Gli1 is distinctly expressed in r-Th, likely under the influence of high levels of Shh, and acts as a transcriptional activator (Ruiz i Altaba, 1998; Mullor et al., 2001; Ruiz i Altaba et al., 2003), as in zebrafish (Karlstrom et al., 2003). Like in mammals, Tcf4 (Tcf7L2) is expressed along the thalamus and habenula and occupies also the Nkx2.2 expressing r-Th. Its expression seems to be mediated by Wnt protein signaling through canonical β-catenin pathway, and its activity is required not only during development but also for the proper maturation and function of the thalamus in adulthood (Bluske et al., 2012). Lhx1 is also expressed in the Nkx2.2 positive cells in *Xenopus* thalamus, contributing to identify the r-Th domain and, like in the mouse, Lhx1 positive cells form very thin bands of one/two-cell diameters in the Zli (Chen et al., 2009; present results).

In the rostral forebrain, the GABA neurons production appeared to be restricted to specific histogenetic territories that expressed Dlx genes, such as the basal ganglia and the prethalamus (Price et al., 1991; Bulfone et al., 1993; Marín and Rubenstein, 2001). However, specific GABA cell populations exist in the thalamus (Dlx-negative) in different vertebrate species (Puelles and Rubenstein, 1993; Martínez-de-la-Torre et al., 2002), indicating that other regulatory genes must influence the acquisition of this phenotype. It has been suggested that the expression of Nkx2.2 in the r-Th leads to the production of the GABAergic cells of the intergeniculate leaflet and other close thalamic areas (Martínez-de-la-Torre et al., 2002; Vue et al., 2007; Jeong et al., 2011; Robertshaw et al., 2013). Actually r-Th neurons express Gad1, encoding glutamate decarboxylase 1, a key enzyme of GABA biosynthesis and a GABAergic differentiation marker (Robertshaw et al., 2013). Our results have shown that early in development, the first GABA cells observed in the thalamus (a region devoid of Dll4 expression) colocalize Nkx2.2 in the r-Th, suggesting this region as the source of the thalamic GABA neurons observed in the mature thalamus (Brox et al., 2003).

### Caudal Thalamic Domain (c-Th)

The specification of the large c-Th domain has been interpreted as the result of signals from the Zli (primarily Shh) that apparently drive the progressive and gradient-dependent downregulation of Pax6 in the thalamus, first in the r-Th and later in the c-Th (Kiecker and Lumsden, 2004, 2005; Vue et al., 2009; Robertshaw et al., 2013; Caballero et al., 2014), although it persists in the habenula and commissural region of the pretectum (Ferran et al., 2007, 2008). In *Xenopus*, a well-established Shh expression in the Zli is not observed before stage 32 (Domínguez et al., 2010), and also before this stage the expression of Pax6 is widespread in the diencephalic alar territories (p1, p2, p3). Starting from stage 32, Shh expression expands dorsally to form the characteristic transverse Zli spike, while Pax6 expression simultaneously diminishes and is progressively limited to the habenula and the pretectal region (present results; Bandín et al., 2014). In turn, Gbx2 (and Dbx1 and Lhx2) expressing cells in the c-Th seem to require low levels of Shh (Szabó et al., 2009; Scholpp and Lumsden, 2010), and their descendents gradually extend dorsally and caudally in the Pax6-free mantle zone of the c-Th and form precise lineage-restriction boundaries that delineate the thalamus from the epithalamus (primordium of the habenula) and the pretectum, like in amniotes (Bulfone et al., 1993; Martínez-de-la-Torre et al., 2002; Chen et al., 2009). The onset of Gbx2 expression in the thalamic neurons appears to be related to the exit of the cell cycle (Bulfone et al., 1993; Nakagawa and O’Leary, 2001; Chen et al., 2009). The key importance of Gbx2 in the development of the thalamus can be inferred by the fact that mice lacking Gbx2 interrupt thalamic histogenesis and suppress almost totally the formation of thalamocortical projections (Hevner et al., 2002; Chen et al., 2009; Chatterjee et al., 2012). In addition, the expression of Gbx2 in mature thalamic neurons was suggested to be necessary for cell survival (Szabó et al., 2009).

Thalamic progenitor cells interpret the Shh gradient regulating the activity of the Gli transcription factors (Lee et al., 1997; Ruiz i Altaba, 1998; Haddad-Tóvolli et al., 2012). In *Xenopus*, we observed that Gli4 (homologous of mammalian Gli2, see Table 2) is expressed caudal to Gli1, in c-Th, where it can act as activator and/or repressor of other genes (Ruiz i Altaba, 1998; Mullor et al., 2001; Ruiz i Altaba et al., 2003). In fact, it has been demonstrated that the low levels of Shh and Gli2 led to Gbx2 expression in the c-Th, in contrast to high levels of Shh and Gli1 that led to Sox14 (and Nkx2.2) expression in the r-Th (Jeong et al., 2011; Suzuki-Hirano et al., 2011). The LIM-homeodomain transcription factors Lhx2/Lhx9 are expressed in the mouse embryonic thalamus (Rétaux et al., 1999; Nakagawa and O’Leary, 2001). The pattern of Lhx9 (and Lhx2) expression in the c-Th of *Xenopus* is similar to that of Gbx2, although it includes the developing habenula (present results; Moreno et al., 2004). It was suggested that Gbx2 and Lhx2/Lhx9 in the thalamus are involved in a common pathway and have a role in the transformation of mitotically active precursors into mature post-mitotic neurons (Hagemann and Scholpp, 2012).

The rostroventral expression of Ngn2 (also known as Ngnr-1 in *Xenopus*; Nieber et al., 2009) and the combination of Ngn2/Dbx1 caudodorsal expression allowed us to distinguish the c-Th1 from the c-Th2 subdomains in *Xenopus* (Figures 8A,B), strictly similar to the situation described in mice, where they were shown to depend on progressively lower amounts of Shh (Hashimoto-Torii et al., 2003; Epstein, 2012). Actually, suppression of Shh expression in the Zli results in the absence of Ngn (and Gbx2) in the thalamus, both in chick and zebrafish (Hashimoto-Torii et al., 2003; Kiecker and Lumsden, 2004; Vieira et al., 2005; Scholpp et al., 2006).

Finally, the patterned c-Th has been demonstrated to give rise to all glutamatergic nuclei of the thalamus, which receive multimodal sensory information that in turn relay to sensory regions of the neocortex, via thalamocortical pathways (Vue et al., 2007; Robertshaw et al., 2013). Thus, the combinations of the genes expressed in this large domain subsequently lead to the observation of high expression of the glutamatergic differentiation marker Vglut2 encoding a vesicular glutamate transporter, as we have also corroborated in the c-Th of *Xenopus*.

### Epithalamus

The epithalamus develops as a derivative of the dorsal alar plate in the caudal part of the diencephalic p2. It gives rise to the epiphysis (pineal complex) and the nuclei of the habenula. It has been generally considered in relation to the thalamus, although with significant developmental differences, and functional characteristics in the mature brain. Because many of the markers used here to characterize the developing thalamus were also observed in the epithalamus we include a few comments on its early genoarchitecture.

During the prepatterning, the early habenular domain is characterized by the combination of the expressions of Dbx1, Irx1, Pax6, and Wnt7b in mice and zebrafish (Chatterjee et al., 2014). Strictly in line with those observations, we have demonstrated that the primordial epithalamus of *Xenopus* acquires its competence by the expressions of similar combinations of markers (Wnt3a, Xiro1, Dbx1, Tcf4, and Pax6; present results) (Figures 7 and 8B,C) and the boundary between the c-Th and the epithalamus was clearly established by mid embryonic stages by the thalamic expression of Gbx2. Comparatively in the mouse embryo, genetic fate mapping studies demonstrated Gbx2 expression in the thalamus and demonstrated that the thalamic Gbx2 lineage marks a clear boundary between the epithalamus and the thalamus (Chen et al., 2009), highlighting a clear segregation of habenular and thalamic neurons. Differently, the LIM genes Lhx9/Lhx2 in *Xenopus* are expressed in parallel to Gbx2 in the c-Th but they are also expressed in the epithalamus, as in fish (Peukert et al., 2011) and mammals (Rétaux et al., 1999). Recent studies have shown that the Lhx9 homeobox gene controls pineal gland development and prevents postnatal hydrocephalus (Yamazaki et al., 2015).

Interestingly, recent studies have shown that Shh has a key role in regulating the specification of the habenula in combination with Pax6, which in turn regulates the temporospatial expression of Shh at the Zli, thus controlling the subdivision of the p2 alar domain into the thalamus and epithalamus (Caballero et al., 2014; Chatterjee et al., 2014). Furthermore, in the alar plate of the Pax6 mutant mouse the epithalamus is missing, whereas the thalamic domain is shifted dorsally (Chatterjee et al., 2014).

### Concluding Remarks

The aim of our study was to analyze the molecular underpinnings of the development of the thalamus in *Xenopus laevis* and evaluate the common traits in vertebrates by comparison with previous data. Our evolutionary developmental (evo-devo) study focuses on a model organism (an amphibian, representative of the only group of anammniote tetrapods) and compares gene expression patterns and molecular profiles that define early brain structures. This is based on the notion that evolutionary close relatives (e.g., vertebrates) share fundamental features of their gene networks during development. In addition, the “same” genomic traits (genomic sequence homology) are rather frequent in related species.

The molecular specification of the rostral and caudal domains in the embryonic thalamus proper was described in great detail in the mouse embryo (Vue et al., 2007; see Puelles and Martínez, 2013), and it was confirmed that orthologous genes are expressed in the counterpart domains of the chicken (Robertshaw et al., 2013), indicating molecular conservation of thalamic patterning in amniotes. In both cases, in the rostral domain originate the GABAergic neurons while glutamatergic neurons derive from the caudal domain.

In this scenario, in the present analysis of the *Xenopus* thalamus we have described and correlated one to another the expression of 22 markers, which serves to highlight that prepatterning and patterning features are largely shared among tetrapods. Furthermore, recent research in the zebrafish diencephalon identified numerous neurological structures and ontogenetic processes as highly conserved across vertebrates (Scholpp and Lumsden, 2010; Mueller, 2012).

## Author Contributions

All authors had full access to all the data in the study and take responsibility for the integrity of the data and the accuracy of the data analysis. AG and RM devised the study. SB performed most of the experiments. The three authors contributed to the data analysis. SB and RM led the figure preparation and wrote the majority of the article, further completed and edited by AG. All authors approved the article.

## Conflict of Interest Statement

The authors declare that the research was conducted in the absence of any commercial or financial relationships that could be construed as a potential conflict of interest.

## Abbreviations

ABB: alar basal boundary
c-Th: caudal thalamus (caudal progenitor domain)
c-Th1: caudal thalamus 1 (caudal progenitor domain 1)
c-Th2: caudal thalamus 2 (caudal progenitor domain 2)
H: hypothalamus
Hb: habenula
mes: mesencephalon
mz: mantle zone
p1-p3: prosomeres 1–3
p2b: p2 basal plate
r-Th: rostral (rostral progenitor domain)
st: stages
tel: telencephalon
Th: thalamus
Tub: tuberal hypothalamus
v: ventricle
vz: ventricular zone
Zli: zona limitans intrathalamica.
